# Master of Metals2: a graph neural network based architecture for the prediction of zinc binding sites in protein structures

**DOI:** 10.1093/bib/bbag078

**Published:** 2026-03-02

**Authors:** Vincenzo Laveglia, Cosimo Ciofalo, Enrico Morelli, Claudia Andreini, Antonio Rosato

**Affiliations:** Department of Chemistry, University of Florence, Via della Lastruccia 3, 50019, Sesto Fiorentino, Italy; Department of Chemistry, University of Florence, Via della Lastruccia 3, 50019, Sesto Fiorentino, Italy; Magnetic Resonance Center (CERM), University of Florence, Via Luigi Sacconi 6, 50019 Sesto Fiorentino, Italy; Consorzio Interuniversitario di Risonanze Magnetiche di Metallo Proteine (CIRMMP), Via Luigi Sacconi 6, 50019, Sesto Fiorentino, Italy; Department of Chemistry, University of Florence, Via della Lastruccia 3, 50019, Sesto Fiorentino, Italy; Magnetic Resonance Center (CERM), University of Florence, Via Luigi Sacconi 6, 50019 Sesto Fiorentino, Italy; Consorzio Interuniversitario di Risonanze Magnetiche di Metallo Proteine (CIRMMP), Via Luigi Sacconi 6, 50019, Sesto Fiorentino, Italy; Department of Chemistry, University of Florence, Via della Lastruccia 3, 50019, Sesto Fiorentino, Italy; Magnetic Resonance Center (CERM), University of Florence, Via Luigi Sacconi 6, 50019 Sesto Fiorentino, Italy; Consorzio Interuniversitario di Risonanze Magnetiche di Metallo Proteine (CIRMMP), Via Luigi Sacconi 6, 50019, Sesto Fiorentino, Italy

**Keywords:** metalloprotein, bioinorganic chemistry, machine learning, structural bioinformatics

## Abstract

Zinc ions play essential structural and catalytic roles in a wide range of proteins. Accurate prediction of their binding sites is crucial for structural and functional annotation. We present MoM2, a web-accessible tool for predicting zinc-binding sites in protein 3D structures. MoM2 employs a graph neural network trained exclusively on spatial features specifically, Cα and Cβ coordinates eliminating the need for templates or sequence-based heuristics. The tool efficiently processes entire proteomes within hours and demonstrates strong predictive performance. In a benchmark of 412 experimentally determined apo-structures, MoM2 outperformed existing methods, achieving the highest F1-score (55.7%) and the lowest false discovery rate (44.1%). The web interface supports input via structure files, PDB or UniProt IDs, and allows batch processing with customizable thresholds. As an independent validation, MoM2 correctly identified 18 out of 20 predicted zinc sites in SARS-CoV-2 proteins. The tool is freely available at https://mom2.cerm.unifi.it.

## Introduction

Zinc is an essential trace element in all domains of life, playing key structural, catalytic, and regulatory roles in a wide range of biological processes [1]. Its functional integration into proteins relies on specific zinc-binding sites, which are characterized by well-defined geometric and chemical properties. Accurately identifying these sites is crucial for understanding protein function, elucidating metal homeostasis, and guiding the design of metal-targeting therapeutics [2, 3].

While experimental techniques such as X-ray crystallography can reveal the presence of zinc ions in proteins, they are often limited by cost, throughput, or sample availability. As a result, computational methods for zinc-binding site prediction have become increasingly important, for example in the context of large-scale protein annotation.

Several tools have been developed to predict metal-binding sites (MBSs), based on either sequence or structure derived information, with varying degrees of accuracy. In the last few years, structure-based tools have been very popular thanks to the availability of reliable structural models provided by DL methods such as AlphaFold [4], RosettaFold [5] or OpenFold [6]. These predictors exploit the knowledge of the spatial arrangement of amino acids to identify potential MBSs. Among structure-based tools a specific category is that of template-based methods. This approach involves comparing a structural model or an experimental apo-structure, which lacks bound metal ions, against a collection of templates. These templates represent known spatial arrangements of potential metal-binding ligands and are typically taken from the Protein Data Bank (PDB) [7] or specialized databases like MetalPDB [8, 9]. The method evaluates how well each template fits the input structure using various criteria [10, 11], such as geometric and biochemical compatibility. When a suitable match is found, the method can identify the likely position of the MBS and, in some cases, even predict the specific type of metal ion that may bind there.

Template-based tools depend on the availability of structurally similar sites to make predictions. If the target protein lacks a suitable template for comparison or if the coverage of the template library is insufficient, the prediction accuracy will be significantly diminished [12]. Machine learning techniques have emerged as powerful tools for the prediction of MBSs in proteins, promising to overcome the above limitations. These techniques utilize curated datasets of metalloprotein structures to train models capable of identifying complex spatial and physicochemical patterns associated with metal coordination. By incorporating features such as residue type, atomic distances, coordination geometry, and electrostatic properties, supervised learning algorithms can generalize to unseen protein structures with satisfactory accuracy [13–15].

In this work, we describe MoM2, a structure-based predictor of zinc-binding sites that leverages machine learning without relying on known metal-binding templates. MoM2 leverages a graph neural network (GNN) architecture that processes the 3D structure of a protein as a graph [16, 17], where each node corresponds to a residue and edges capture spatial relationships. This design enables MoM2 to learn complex spatial patterns associated with zinc coordination, and to generalize beyond previously characterized metalloproteins. We benchmarked this tool using experimentally determined apo-structures. We then demonstrated its applicability at the proteome scale. Our results demonstrate that MoM2 delivers state-of-the-art performance, scales efficiently to large and complex systems, and offers a user-friendly interface through a dedicated web server (https://mom2.cerm.unifi.it), making it a valuable tool for the functional annotation of metalloproteins.

## Methods

### Training set

The training dataset was the same we used to develop Master of Metals [18]. It consists of a list of target metal-binding sites (MBSs), identified by the format <pdb_code>_<site_index>. The MBS is a substructure that surrounds the metal ion(s) and represents the macromolecular environment of the metal. It may be automatically derived from the 3D structures in the Protein Data Bank (PDB) [7]. This substructure should match the minimum environment that governs the metal's functionality, also known as the "minimal functional site" [19]. By construction, each experimental MBS is associated with a PDB structure that contains it.

In this work, we addressed only zinc-binding proteins but for simplicity we maintain the more general MBS wording. For PDB structures containing multiple MBSs, only a subset may be included in the dataset, e.g., when identical sites are present in symmetrical or repeated subunits. In the present work we used only sites with at least three residues. Dinuclear sites containing zinc along with a second, different metal were also excluded. The final dataset comprises 1294 structures with 1803 target sites.

### Independent test set

To prepare an independent dataset for the validation of the performance of MoM2, we queried MetalPDB for all sites containing zinc(II) ions and then removed from this list all those contained in the training dataset. The resulting list includes both physiological and non-physiological sites; the latter are sites detected in X-ray structures that are populated because of the experimental conditions required for protein crystallization and are not functionally relevant in cells. We expect non-physiological sites not to be of interest to the users, as they do not contribute to cell functioning, thus they were discarded based on the available annotation in MetalPDB. The list contains chains that are homologous to those in the training set, which must be removed. To address this, we labeled all the sequences in the training set as "Train" and those associated in the second dataset as "Independent". The sequences of both groups were clustered together at 30% identity using CD-HIT [20] and only the clusters containing exclusively "Independent"-tagged chains were retained. From these, we kept only the clusters with at least three sequences, as clusters with more sequences are more likely to be physiological. Each of these clusters was then associated with zinc(II)-binding patterns (e.g., CX(2)CX(12)HX(3)H) from MetalPDB. We retained only the clusters that had at least one pattern with three or more ligands of type Cys, His, Asp, or Glu (the so-called CHED group [21, 22]), which are more likely to be physiological [23]. Since different patterns can be associated with the same cluster, to reduce redundancy we kept only the sites that differed in their ligand string (e.g., "HHH" vs "HCHC"). This means that within the same cluster, patterns like HX(3)HX(5)H and HX(22)X(83)H are considered equivalent because they both generate the string "HHH", and thus only one of the two sites is retained. We then used the stand-alone version of FoldSeek [24] to perform a structural comparison between all chains in the training set and those in the test set and to remove from the latter any structures that featured an e-value < ${10}^{-5}$ with at least one structure in the training set. With this procedure, 60 chains were discarded, and the tool’s performance was recalculated on the remaining 312 structures.

### Description of the workflow

Protein structures are represented as graphs, where each node corresponds to the Cα atom of a residue, and an edge between two nodes *i* and *j* exists if the distance between their Cα atoms is less than a certain threshold. In this work we used a 11.5 Å threshold, on the basis of the results obtained varying the value from 10.0 to 12.0 in 0.5 Å intervals. Similar ranges have been explored in other publications leveraging graph representations e.g. to predict enzyme functions [25]. Each node of the graph is associated with a one-hot vector, with a value of 1 at the position corresponding to its amino acid type. Each structure is associated with a tensor *R* of size *L×L×*2, L being the protein sequence length, which can be thought of as an *L×L* matrix where each element is a two-dimensional vector. The element at position (*i, j*) contains the distances between Cα_i_–Cα_j_ and Cβ_i_–Cβ_j_ of the two associated residues. Gly residues were not included in the graph representation as they do not have a Cβ atom.

The first step in the MoM2 workflow is the classification of each individual residue in the structure (i.e., each node in the graph) as either belonging or not belonging to an MBS. To process these graphs, a Message Passing Neural Network (MPNN) has been implemented. This type of neural network is particularly well-suited for graph-structured data, as it can incorporate features not only from the nodes but also from the edges. The model is composed of two MPNN layers, each having dimensionality of 70 neurons. After the first MPNN layer, a Layer Normalization step is applied. For each protein structure, the network takes as input the data described in the previous step. The output is a matrix Y of size *L×2*, where the *j*-th row corresponds to the prediction for the *j*-th residue. If the first element of the row is lower than the second, the residue is predicted to be part of an MBS; otherwise, it is not. The prediction for each residue is influenced not only by its own features but also by the features of its spatially close residues and the relational features encoded in the tensor *R*.

Once we have identified which residues are likely to be part of a site, the next step is to organize them into MBSs. The underlying assumption is that a residue belongs to a site if and only if there are at least two other predicted metal-binding residues in its vicinity that meet specific geometric criteria. To implement this step, we adopted the procedure of the BioMetAll algorithm [26]. The 3D space around the protein is filled with probe atoms, forming a kind of 3D grid. For each probe, measurements are taken relative to all the predicted CHED residues from the previous step. Each probe is then associated with the residues that satisfy certain structural restraints involving distances and angles [26]. If a probe is associated with at least three predicted residues, those residues are considered to form a potential MBS. Multiple probes may be associated with the same set of residues, effectively identifying the same site. As a result, we transition from having a list of individual metal-binding residues to a list of candidate MBSs. In each candidate MBS the position of the zinc(II) ion is estimated by averaging the position of the probes that identified it.

To define a metric for evaluating the quality of the predictions and rank them, we processed the sites in terms of residue pairs. This choice was based on the observation that two residues belonging to the same site tend to be arranged in a specific spatial configuration, particularly with respect to the directionality of the Cα–Cb vectors, which depends on the specific pair of residue types being analyzed. For this reason, a separate analysis was conducted for each possible type pair of the CHED group (e.g., His-His, His-Glu, …, Cys-Cys, …). Thus, we trained 10 feedforward neural networks (FFNNs) to predict whether, based on their spatial arrangement, a pair of residues is indeed likely to belong to an MBS. For a given residue pair (Ri, Rj), the outcome depends upon the identity of residues *i* and *j*, the distance between their Cα atoms, the distance between their Cb atoms, the angle formed between the vectors (Ca_i_, Cb_i_) and (Ca_i_, Ca_j_), and the angle between the vectors (Ca_j_, Cb_j_) and (Ca_j_, Ca_i_).

For each predicted MBS, the set of 10 FFNNs is used to determine the positivity or negativity of all the relationships (namely, residue pairs) it comprises. Thus, a site is conceptualized as an ensemble of relationships. Ideally, all relationships within a site should be predicted as positive. However, due to their structural heterogeneity and the fact that some amino acid pairs are much rarer than others, not all pairs are predicted equally well (Table S1). In the majority of cases the recall ranged from 77% to 99%, whereas the FDR was between 1% and 24%. The pair Cys-His proved to be the most difficult to assess, with a recall of 67% and a FDR of 24%. Based on a non-redundant dataset from MetalPDB, Cys-His pairs are present in 31% of zinc(II) MBSs. Given this frequency and the recall rate discussed above, we estimate that roughly one in five sites may contain a valid Cys–His relationship that our classifier will fail to detect.

For these reasons, we do not impose that all relationships in an MBS are predicted as positive. Indeed, a custom scoring function was implemented to evaluate predicted sites. We define *n_valid_* as the number of relationships predicted as positive and *c_max_* as the highest confidence score among those positive predictions. Then our scoring function is


$$ Score=\frac{1}{2}\ \frac{n_{valid}}{4}+\frac{c_{max}}{2} $$

Considering that the number of relationships in a site is *N(N*-1)/2, with *N* being the number of residues in the site, and that *c_max_* can be 1.0 at most, the score is dependent on the number of residues in the site. The highest score can be 0.875 for sites with three residues, 1.25 for sites with four residues and > 1.5 for sites with five residues or more. This is intentional, as we want to favor larger predicted sites in our final ranking. In practice, for a given *c_max_*, the scoring function depends solely on the *n_valid_* parameter and is therefore independent of the total number of residues in the MBS (which in reality is not known). In our approach, MBSs are first built with the BioMetAll algorithm, then the pairwise relationships within the site are individually assessed by the 10 networks. As a result, it is possible that, for example, in a site comprising four residues, only three of the six relationships are deemed valid (i.e. *n_valid_* =3, yielding a score of 0.875 if *c_max_* = 1.0). Importantly, when a binding pair is identified with high *c_max_*, it serves as an anchor for the scoring function, enabling smaller sites to surpass the threshold. Our strategy aims at identifying enough residue relationships to confidently establish the presence of the MBS. By setting the normalization factor of the *n_valid_* term to the fixed value of 4 we tune the score so that any site with at least four valid relationships can have a score of 1.0 or higher, if *c_max_* = 1.0. Conversely, normalizing by the total number of possible relationships, *N(N*-1)/2, would implicitly require sites with more ligands to have a proportionally larger of valid relationships. Given the limitations of the FFNNs discussed above, this appeared difficult to achieve in practice. Consequently, such an approach would have biased the predictor toward smaller sites over larger ones, which is undesirable. At the same time, our choice of the fixed normalization factor slightly disfavors MBSs with three ligands.

Multiple predictions may correspond to the same site, with each prediction capturing only a subset of the target residues and occasionally incorporating nearby non-target residues. To address this redundancy, an aggregation procedure was implemented. For any pair of predicted MBSs, the inter-residue distances are computed; if the maximum distance between residues is less than 3 Å and the residues of one site are not entirely contained within the other, the sites are merged. This iterative process continues until no further aggregations are possible. Following aggregation, the score of the site is recomputed. Finally, any site for which more than 50% of its residues are already present in a higher-scoring site is excluded from the final output. The estimated position of the zinc(II) ion is then recalculated as the average of the individual positions in the merged MBSs.

### Training

The MoM2 pipeline contains three main modules. The first module performs the prediction of individual metal-binding residues, where each residue is classified as non-binding (0), nearby [1], or binding [2]. The binding residues [2] are protein amino acids that contain at least one non-hydrogen atom at a distance smaller than 3.0 Å from the metal. Nearby residues [1] are identified as those that possess at least one atom located within 5 Å of a binding residue. All other residues are labeled as non-binding (0). This information is obtained directly from MetalPDB contents. This first module implements a Message Passing Neural Network (MPNN), which processes protein structures represented as graphs. The network is trained to assign a class to each node in the graph using a dataset split into training, validation, and test sets in an 80:10:10 ratio. Training proceeds for up to 1000 epochs, with early stopping applied if no improvement is observed over 10 consecutive epochs. The model is then evaluated on the test set. The training uses a learning rate of 0.001, and a maximum of 500 epochs. A custom loss function was employed as follows: for each input structure, the loss is calculated as the sum of the Mean Squared Error losses over the points (residues) of each individual class (0, 1, 2). Additionally, the loss for class 0 was weighted by a factor of 1.5. Hyperparameters such as architecture size and learning rate have been optimized through random search [27].

The second module defines the binding site based on geometric constraints extracted from the literature [26]. This module does not require any training as it uses pre-defined rules with known cut-off values.

The third module assesses the predicted MBSs. For this, ten neural networks, each responsible for predicting whether a specific amino acid pair belongs to a binding site, were trained. For each pair, a sub-dataset was generated from the entire Training dataset by taking the relevant instances. In detail, all known sites from the training set were collected, along with all per-residue predictions generated previously (note that the MPNN used in the first module was trained separately). For each predicted MBS, all possible residue pairs were generated. If both residues in a pair were true target residues, the pair was labeled as positive; if at least one residue was not a target, the pair was labeled as negative. The resulting dataset was then separated by pair type and randomly split into training, validation, and test sets. This dataset was used to train a two-layer Multilayer Perceptron (MLP) to predict whether a given residue pair is positive or negative. Each MLP receives four input features (only relation features, as residue identities are now constant), and consists of two hidden layers with either 200, 150 or 100 neurons, using layer normalization, ReLU activation, and a dropout rate of 0.01. Training was conducted over 500 epochs with minibatches of 64 elements, evaluating performance every 10 epochs and saving the best-performing model based on validation results. As with the MPNN, the hyperparameters for the multilayer perceptrons have been determined via random search. The output layer has two neurons: one for the negative class and one for the positive class. The predicted class corresponds to the neuron with the highest output value, while the confidence of the model was defined as the absolute difference between the two outputs. A total of ten MLPs were trained, one for each possible pair of CHED residues.

### Positioning the metal

The initial position of the metal ion is computed as the average position of the probes placed by the BioMetAll algorithm. If at least one atom not belonging to a ligand residue lies within 2 Å of the metal, the position is recalculated by selecting, among the probes located at least 1 Å away from all atoms of non-ligand residues, the one closest to the initial metal position. If no probe meets these criteria, the initial metal position remains unchanged.

## Results and discussion

The MBS is a substructure that surrounds the metal ion(s) and represents the macromolecular environment of the metal. In this work, we used the definition of MBS implemented in MetalPDB [9]. In this work, we developed a novel machine learning tool for predicting zinc-containing MBSs in 3D protein structures.

### Architecture of MoM2

**Figure 1:**
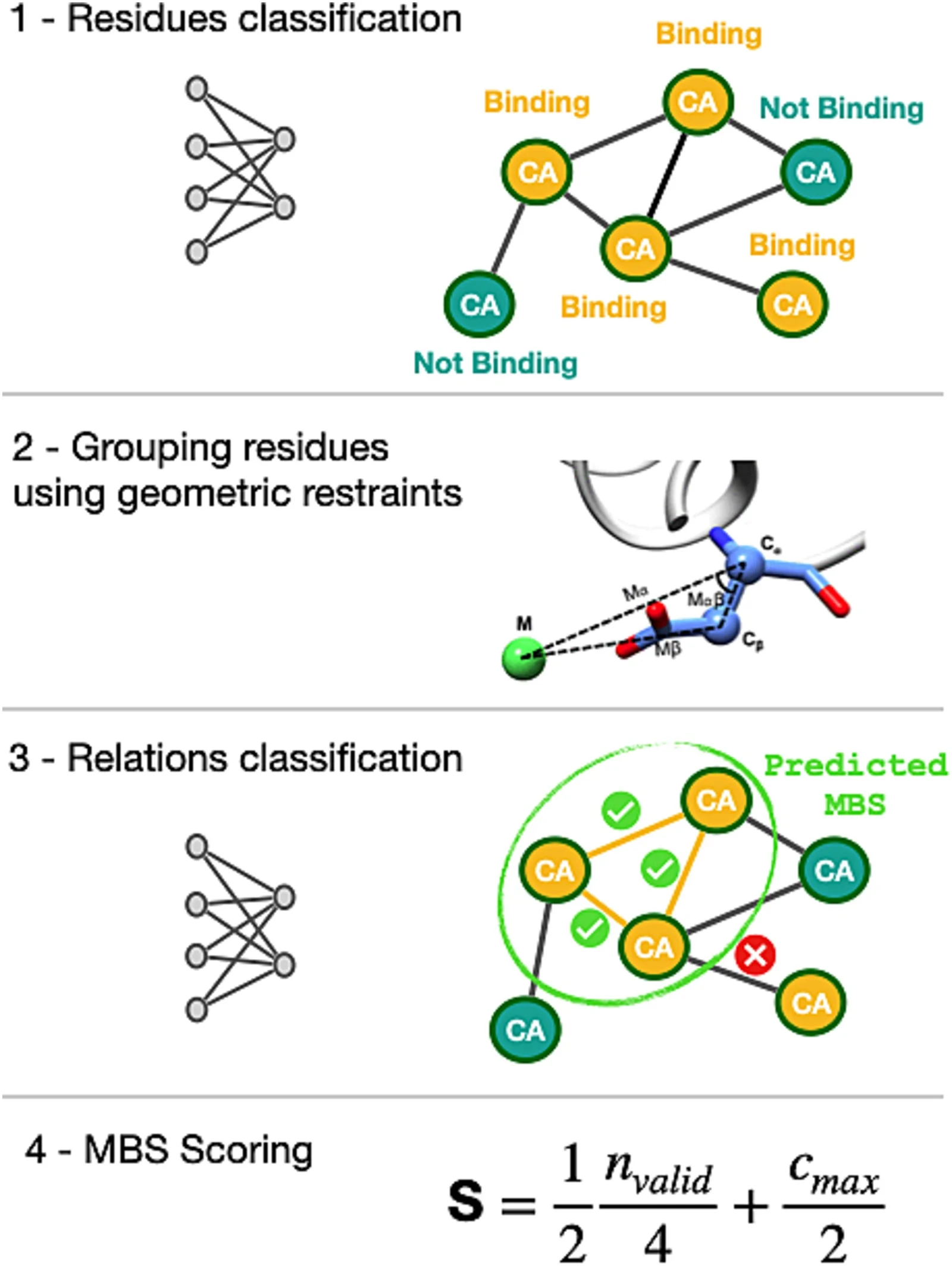
Workflow of MoM2. In the first step, starting from the 3D structure of a protein, a graph is generated, where the nodes represent the amino acid residues. This graph is then fed into a graph neural network, which classifies each node as either binding or non-binding. In the second step, the residues classified as binding are grouped based on geometric constraints, forming coherent sets that represent potential MBSs. Next, the residues within each site are analyzed in pairs. For each pair, relevant structural features are extracted and processed by a feedforward neural network to predict the validity of the interaction. Less probable interactions are discarded. Finally, each identified MBS is assigned a score using a dedicated scoring function.

Master of Metals 2 (MoM2) leverages only geometric features of the protein structure, namely the coordinates of Cα and Cβ atoms. More in detail, we employed a graph-based deep learning pipeline. Protein structures were represented as graphs, where nodes correspond to residues and edges reflect spatial proximity. A Message Passing Neural Network (MPNN) was trained to classify individual residues as zinc-binding or not (Figure 1). The predicted binding residues were then grouped into candidate MBSs using geometric restraints inspired by the BioMetAll algorithm [26]. These restraints include distances and angles between the Cα-Cβ vectors of different residues. To reduce redundancy, overlapping predictions were aggregated, and only high-confidence, non-overlapping sites were retained. Finally, each candidate site was evaluated using a custom scoring function based on the spatial features of pairwise residue relationships (Figure 1). This approach enabled robust identification and ranking of potential zinc-MBS across diverse protein structures, even in the absence of structurally similar template sites. It is important to remember that any ML model performs best on inputs that resemble those seen during training. Consequently, MoM2 is expected to yield reliable predictions for stably folded globular proteins, whereas its performance is likely to degrade for proteins that are intrinsically disordered or otherwise far from the training distribution.

Besides the completely changed ML architecture, the main practical difference between the present tool and its predecessor is its full independence from structural templates. Based on our tests on a workstation equipped with an Intel i7-13700 CPU with 32 Gb of RAM and a GPU RTX 4070 with 16Gb of RAM, MoM2 can process entire proteomes within a few hours, thanks to its efficient design.

### Performance

MoM2 was trained using the same dataset as MoM [18]; for training purposes the latter included only holo sites. In addition, a new dataset (test dataset) was generated to have an independent evaluation of the performance of the tool.

**Figure 2:**
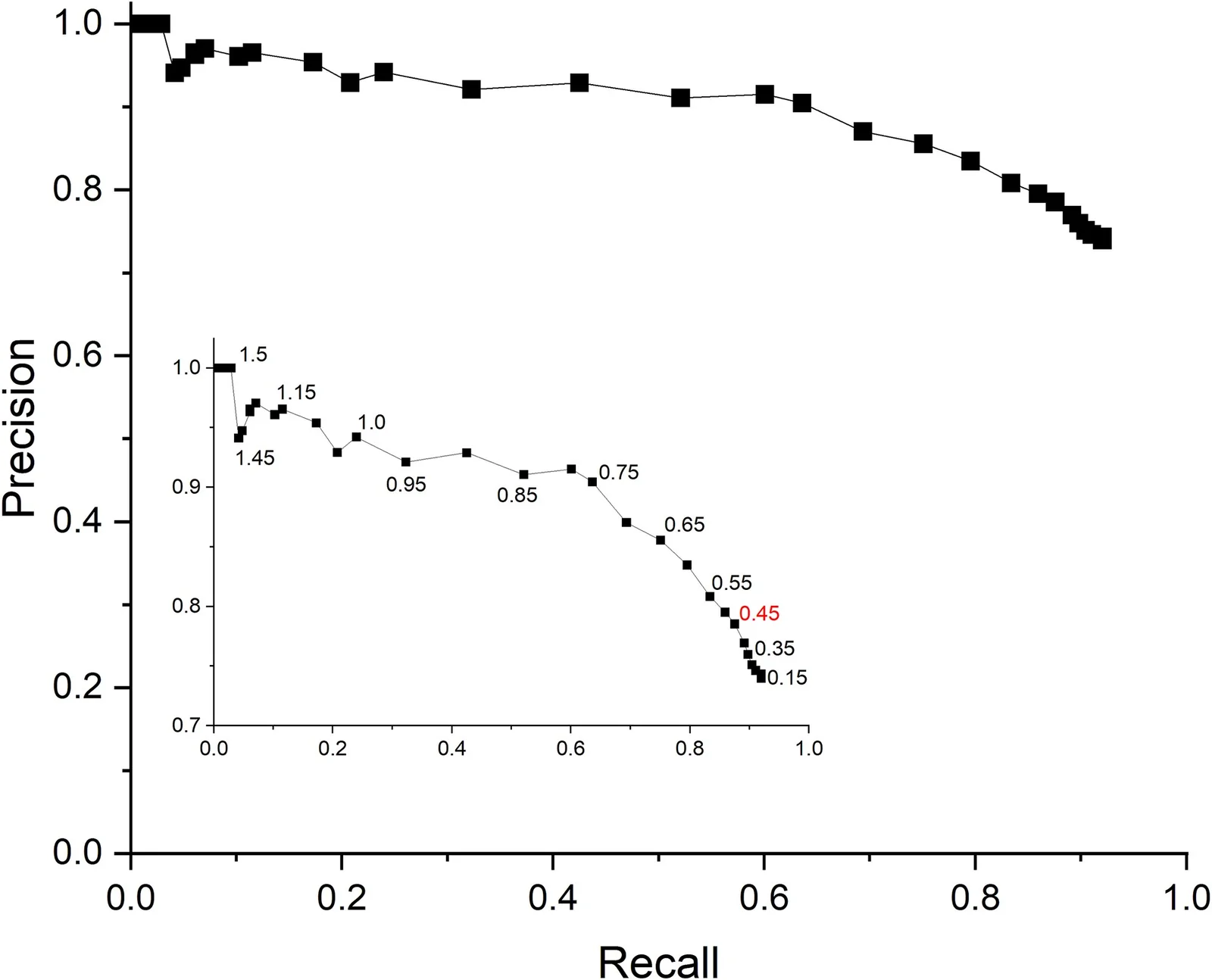
Precision vs recall for predictions computed on the chains containing the target sites. Each point is associated with its corresponding scoring function value. Within the figure, a zoomed-in view of the results is shown, focusing on the precision range from 0.7 to 1.0. The scoring function value corresponding to the maximum F1-score is highlighted in red.

A prediction is considered a correct match (i.e. a True Positive, TP) if it shares at least three residues with the target site. For targets that consist of only three residues, identifying at least two of them is sufficient for a correct match. Only one TP is recorded per target site, regardless of how many matching predictions are output by MoM2. To identify false positives (FPs), we considered all predicted sites generated for a given input structure that did not correspond to any experimentally validated MBS. To calculate the recall, the total number of TPs is divided by the total number of target sites. Precision is calculated by dividing the number of TPs by the total number of predictions output by MoM2. This approach accounts for the possibility that multiple predictions may correspond to different parts of the same site. Since these predictions can all be valid, the computed precision includes all of them. Figure 2 reports the recall and precision values as a function of score threshold. To evaluate the performance of the tool, the precision-recall (PR) curve was calculated using the structures included in the test set. The analysis was carried out in two distinct scenarios: in the first, only the individual chains containing the target sites were used (Figure 2); in the second, complete multimeric structures were used as input (Supplementary Figure S1). In both cases, the PR curves are quite satisfactory. In the first scenario, a precision of 65% is achieved with a recall around 90%, while in the other, precision never drops below 70%. Both curves display a very smooth variation of precision as a function of recall.

### The MoM2 web server

**Figure 3:**
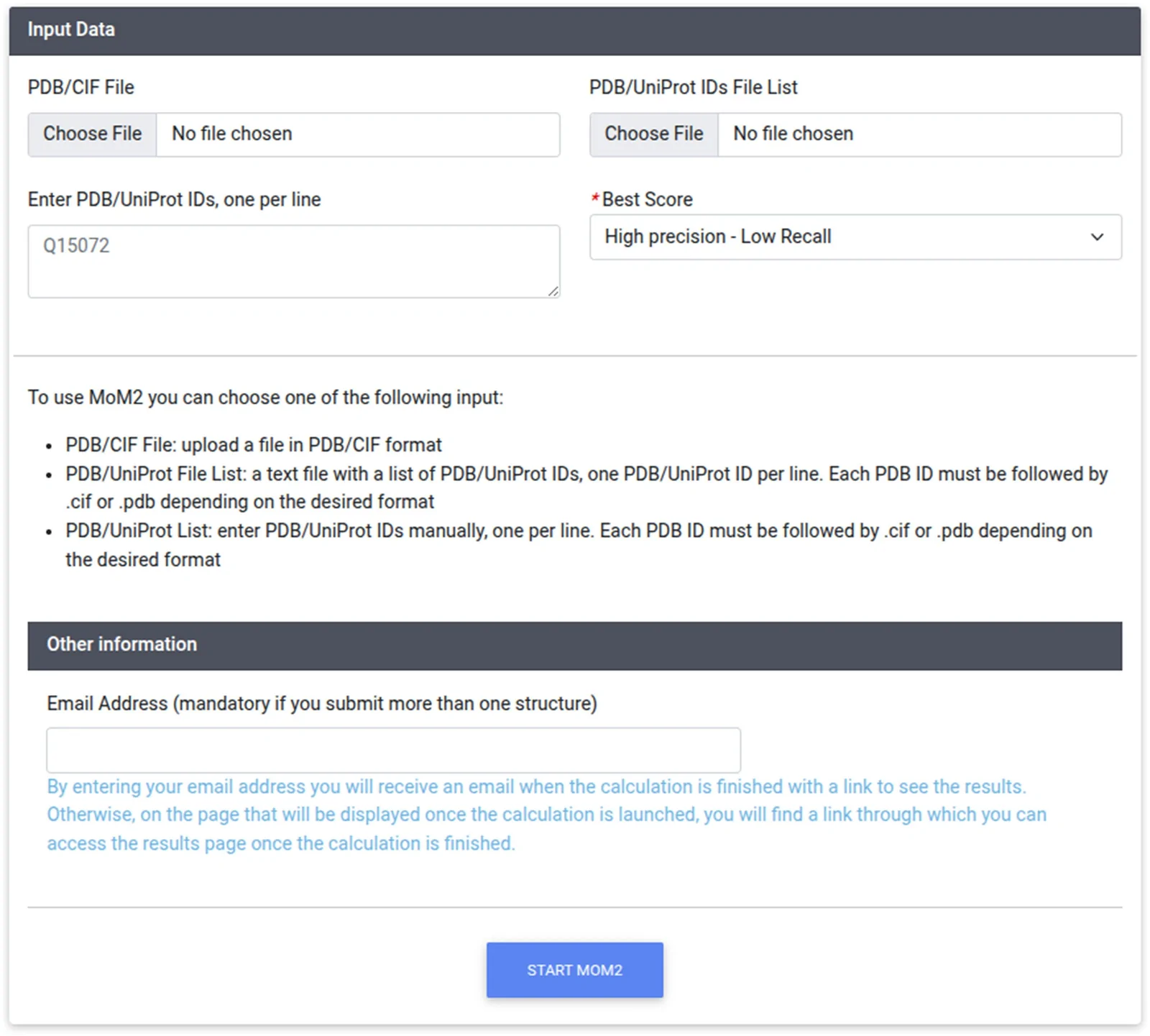
Interface of the web server (https://mom2.cerm.unifi.it/).

To boost the availability of MoM2 for the scientific community, we provided a web server to use our tool (Figure 3). The interface of the servers allows the user to provide their input by uploading a single structure file in the legacy PDB or mmCIF formats, by specifying the PDB ID of an already deposited structure, or by providing the UniProt ID of the protein(s) of interest [28, 29].In the latter case, the model of the 3D structure of the protein is automatically downloaded from the AlphaFold2 database [4]. It is also possible to upload a file containing a list of IDs, one per line. All IDs should be of the same type. Finally, the interface allows the user to set a qualitative threshold for the predictions to be retained and displayed in the output page. Three levels are possible: high precision/low recall; medium precision/medium recall; low precision/high recall. Practically, this choice corresponds to selecting three different threshold values for the scoring function, namely 0.75, 0.45 and 0.15, respectively. The interface provides the option, which is not mandatory, to input an email address to receive notification of calculation completion. For a single monomeric or dimeric protein structure, the calculation time is typically below 1 minute. The code of MoM2 is available also through our Github repository (@cerm_cirmmp; github.com/cerm-cirmmp/MOM2).

The output (Supplementary Figure S2) consists of a Table listing the MBSs predicted for each input protein. Each site is assigned its own specific score, which can be examined in the linked pop-up table. The complete output can be downloaded as a compressed zip archive; the latter includes a structure file with the coordinates of the predicted zinc ions. Note that by design MoM2 predicts the identity of the protein residues serving as metal ligands, and the position of the metal is derived from the latter information.

### Benchmarking on apo-structures

At present there are many different tools that can predict MBS with good to excellent performance reported in their respective publications [18, 26, 30, 31]. Thus, we want to have an overview of the performance of MoM2 with respect to that of the other available tools. For this, we adopted the benchmark previously developed by some of us [32]. This data set includes 412 experimental structures of zinc-binding proteins in their apo form, i.e. structures that were experimentally determined in the absence of the metal cofactor. As each structure can harbor multiple sites, the number of MBSs in the benchmark is 840. This dataset is challenging for the predictors, because of the extensive structural rearrangements that occur upon zinc(II) binding in the site [33]. This benchmark was designed to include only positive cases, hence the present analysis focused exclusively on positive predictions. The results are summarized in Table 1 and Supplementary Figure S3.

**Table 1:** The columns in the table, from left to right report the name of the predictor used (tool), the total number of outputs generated by the predictor for the benchmark (N. Outputs), the total number of the processed structures (N.Structures), the positive predictive value (PPV), which equals TP / (TP + FP), the false discovery rate (FDR), which equals FP / (TP + FP), the recall, which equals TP / (total n. of available sites in the dataset) and the F1-score, which equals 2 × (PPV × recall) / (PPV + recall).

Tool	N. outputs	N. Structures	PPV(%)	FDR(%)	Recall (%)	F1-score
MoM 2	819	389	55.9	44.1	55.5	55.7
BioMetAll	2032	408	9.6	90.3	23.9	13.7
Metal1D	844	385	15.5	84.9	15.5	15.2
GASS	2055	411	19.4	80.6	48.6	27.6
MoM	693	238	53.7	46.3	45.4	49.2
Metal3D	503	239	48.9	51.1	29.8	37.1

For each tool, we took into account at most five output predictions for each input structure, provided the corresponding scores were better than the threshold recommended by the authors. As discussed previously, this approach provides the optimal balance between the total number of sites in the benchmark and the number of predictions evaluated per structure [32]. Note that some tools may provide less than five predictions for a given input, particularly if they have a threshold for the quality of the output. First of all, it must be noted that out of the 412 structures of the benchmark, MoM and Metal3D output predictions only for about 58% of the dataset. Instead, the other four tools could handle between 93.4% and 99.8% of the input structures. This may be due to the software crashing for a specific input, such as in the case of the single structure for which GASS did not produce predictions, or because no predictions with a score better than the threshold were computed. We conducted the present analysis after filtering out redundant true positives, i.e. multiple, slightly different predictions output for the same site in each structure, for all predictors. To do so, for every correct prediction sharing n − 1 ligands with the known holo-site (where n is the total number of ligands), we kept only the highest-scoring prediction. This procedure mirrors a user’s approach of treating any predictions that share all but one residue as identifying the same site.

MoM2 achieved the highest PPV, highest Recall and lowest FDR with respect to all other tools in the analysis, using the most stringent 0.75 threshold value. As observed previously, Metal3D has a satisfactory performance in terms of PPV, whereas its Recall is limited presumably to its lower tolerance to structural rearrangements. MoM2 improved upon MoM particularly for its higher Recall (55.5 % vs 45.4%). This is directly linked to the capability of MoM2 to generate an output for a larger number of the apo-structures of the benchmark (389 vs 238, corresponding to 819 vs 693 sites, respectively). The tool with the worst FDR was BioMetAll, because it produced multiple predictions for each input structure, the majority of which were wrong. Similarly to what we hypothesized for MoM, the good PPV of MoM2 on apo-structures is likely attributable to its algorithmic design, which relies solely on the coordinates of Cα and Cβ atoms. Indeed, these atoms tend to undergo less positional variation between apo and holo forms compared to side chain atoms [33]. The F1-score is the harmonic mean of PPV and Recall. It symmetrically represents both measures in one metric that summarizes the predictive performance. MoM2 achieved the highest F1-score of all tools (55.7%), followed by MoM (49.2%) and Metal3D (37.1%).

Given the potential for large-scale proteome analysis, we measured the average time required by the algorithm to generate a prediction (Table 2). MoM2, MoM and Metal1D featured the shortest calculation times. All the calculations were performed on local installations except for GASS, which can be run only on the web server.

**Table 2:** Average calculation time to produce an output, based on the data for 100 proteins randomly selected from the apo-structures benchmark. The workstation used for the computations was equipped with an Intel i7-13700 processor, 32 GB of RAM, and an RTX 4070 GPU with 16 GB of memory.

Predictor	Average time per output (min)
MoM2	<1'
GASS	5'
MoM	<1'
Metal3D	13'
BioMetAll	4'
Metal1D	<1'

### SARS-CoV-2 predictions

Structural biology studies on severe acute respiratory syndrome coronavirus 2 (SARS-CoV-2) have shown that zinc(II) is a commonly bound metal ion in viral proteins [34–37]. Based on this evidence, we decided to test our predictor on previously proposed zinc(II)-binding proteins from SARS-CoV-2, based on a sequence-based predictor [35]. To compare our structure-based predictions with the previous results, here we took as input all the NSP proteins from NSP1 to NSP16 plus ORF3a. We used experimental structures when their structural coverage was at least 98% of the whole sequence; otherwise, the structures were generated from the sequence using the AlphaFold3 server [38]. Out of the selected proteins, seven were previously predicted to harbor a total of 20 MBSs [35], of which 17 are experimentally validated. Only two coronaviral proteins were included in the training set of MoM2, containing three distinct sites.

**Figure 4:**
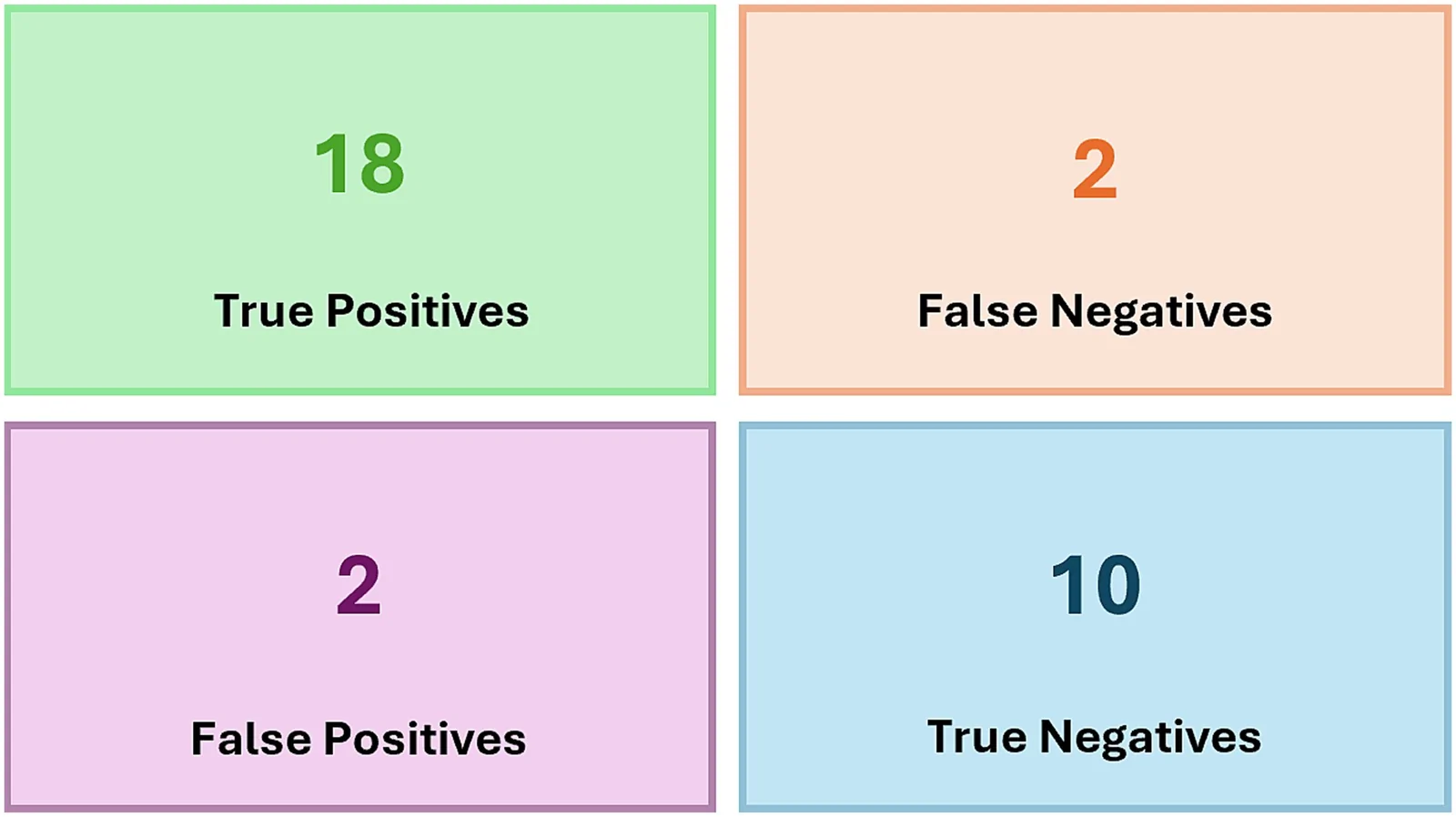
Confusion matrix of MoM2 for the prediction of zinc(II)-binding proteins in SARS-CoV-2 vs a previous dataset of SARS-CoV2 zinc-proteins output by a sequence-based predictor.

The results generated are summarized in Figure 4. MoM2 predicted 18 out of the expected 20 zinc sites, while generating 2 false positives. This corresponds to a recall of 90% and an FDR of 10%. Upon exclusion of the three sites already present in the training dataset, these figures become respectively 88.2% (15 TPs for 17 expected sites), and 11.8%.

### Application of MoM2 to the complete human and Escherichia coli proteomes

Current predictors for zinc-binding sites have reached a high level of reliability [32]. In particular, high performance is obtained when AlphaFold2-generated structures are used as input, likely as a result of the more accurate orientation of side chains in the model than in experimental apo-structures [33]. Notably, the present work focuses on physiologically relevant sites binding a single zinc(II) ion, presumably with a significant degree of pre-organization, making it easy for AlphaFold to predict accurately a conformation quite close to the metal-bound state. The situation may be different for sites binding complex, possibly flexible, organic molecules, especially when the free and bound states are not equally represented in the PDB [39, 40]. These findings support the use of structure-based predictors on a proteome-wide scale, which can provide broader insights into zinc utilization across different organisms. To evaluate the use of MoM2 in this context, we applied our tool to the complete proteomes of *Escherichia coli* and *Homo sapiens*. These two organisms were selected as representative examples of prokaryotic and eukaryotic systems, and because their proteomes are well annotated. All input structures were downloaded from UniProt and restricted to models generated by AlphaFold2, ensuring uniform structural quality and mimicking the common situation in which no experimental coordinates are available. For these UniProt entries, we flagged every protein already annotated as zinc-binding. We included 139 zinc-proteins for *E. coli* and 243 for the human proteome. The analysis was carried out under stringent conditions: only the best prediction output by MoM2 was considered, provided that its score met or exceeded the set threshold. A prediction was considered a true positive if at least one high-confidence site was predicted in a protein annotated as zinc-binding. Conversely, the annotated zinc-proteins for which MoM2 did not produce any valid prediction were counted as false negatives. The results obtained are summarized in Table 3. The recall was similar for both proteomes at the most stringent 0.75 threshold, whereas it was modestly better for *E. coli* at the looser threshold values. The results of Table 3 are slightly better than what expected based on the PR curve (Figure 2), and provide a qualitative indication that the scoring procedure overall captures well the correct MBSs despite some residue pairs, such as the fairly common Cys-His, are ranked worse than others.

**Table 3:** Number of true positives (TP), false negatives (FN), and corresponding recall values for MoM2 predictions on the *Escherichia coli* and *human* proteomes with different thresholds.

Organism	Threshold	TP	FN	Recall (%)
*Escherichia coli*	0.75	106	33	76.3
	0.45	130	9	93.5
	0.15	132	7	95.0
*Human*	0.75	187	56	76.9
	0.45	221	22	91.0
	0.15	227	16	93.4

## Conclusions

In this study, we introduced MoM2, a novel structure-based tool for the prediction of zinc-binding sites in proteins. Unlike its predecessor, which relies on template-based strategies and requires the knowledge of structurally similar sites to make a prediction, MoM2 leverages a deep learning framework based on graph neural networks and geometric features extracted directly from protein structures. This design enables MoM2 to detect MBSs even in the absence of similar templates, overcoming a key limitation of template-dependent methods. MoM2 was compared against other state-of-the-art structure-based predictors using a benchmark dataset of experimentally determined apo-structures. In this challenging setting, MoM2 achieved the highest F1-score among all tools tested.

As a further study case, we analyzed the SARS-CoV-2 proteins, several of which had been proposed to bind zinc(II) ions based on previous sequence-based predictions [35]. Applying MoM2 to the full set of viral non-structural proteins (NSPs) and ORF3a, the tool successfully recovered 18 out of 20 expected zinc-binding sites, with a false discovery rate of just 10%.

Importantly, MoM2 also proved effective at the proteome scale. We applied the model to the full *E. coli* and human proteomes using AlphaFold2-generated structures as input and then assessed its predictive performance by comparing the results to the subset of proteins annotated as zinc-binding in UniProt. This analysis confirmed MoM2’s predictive power and practical utility in large-scale settings. These results underscore its potential to uncover novel metalloproteins and to support functional annotation efforts across diverse organisms.

To maximize accessibility, MoM2 has been made available via a user-friendly web interface, supporting both single-structure and batch predictions.

**Key Points**

- Biological relevance: Accurate identification of zinc-binding sites in proteins is essential for understanding protein function, metal homeostasis, and the development of metal-targeted therapeutics.
- MoM2, a deep learning–based solution: MoM2 is a novel structure-based predictor that employs a Graph Neural Network (GNN) to identify zinc-binding residues directly from 3D protein structures, without relying on known template sites.
- Benchmarking and performance: MoM2 outperforms existing state-of-the-art tools on challenging test sets, including experimentally determined apo-structures. It achieves high precision and F1-scores, demonstrating robustness to conformational variability.
- Scalability and efficiency: Thanks to its computational design, MoM2 can process entire proteomes in a matter of hours, making it suitable for large-scale annotation tasks.
- Accessible via web server: A user-friendly web interface (https://mom2.cerm.unifi.it/) allows users to input protein structures, PDB IDs, or UniProt IDs. Results include ranked predictions with interactive visualizations and downloadable output files.

## Supplementary Material

revised_supplementary02_bbag078

## Data Availability

The code of MoM2 is freely available at https://github.com/cerm-cirmmp/MOM2. MoM2 is available as a web server at https://mom2.cerm.unifi.it/; no registration is required.
