# Supplementary material for: Master of Metals2: a graph neural network based architecture for the prediction of zinc binding sites in protein structures
Source: Brief Bioinform. 2026 Mar 2;27(2):bbag078. doi: 10.1093/bib/bbag078 (PMC12951075; doi:10.1093/bib/bbag078)
Supplement: revised_supplementary02_bbag078 [file revised_supplementary02_bbag078.docx]

**Master of Metals2: A Graph Neural Network Based Architecture for the Prediction of Zinc Binding Sites in Protein Structures**

Vincenzo Laveglia^1^, Cosimo Ciofalo^1,2^, Enrico Morelli^3^, Claudia Andreini^1,2,3,*^, Antonio Rosato^1,2,3,*^

**Supplementary Material**


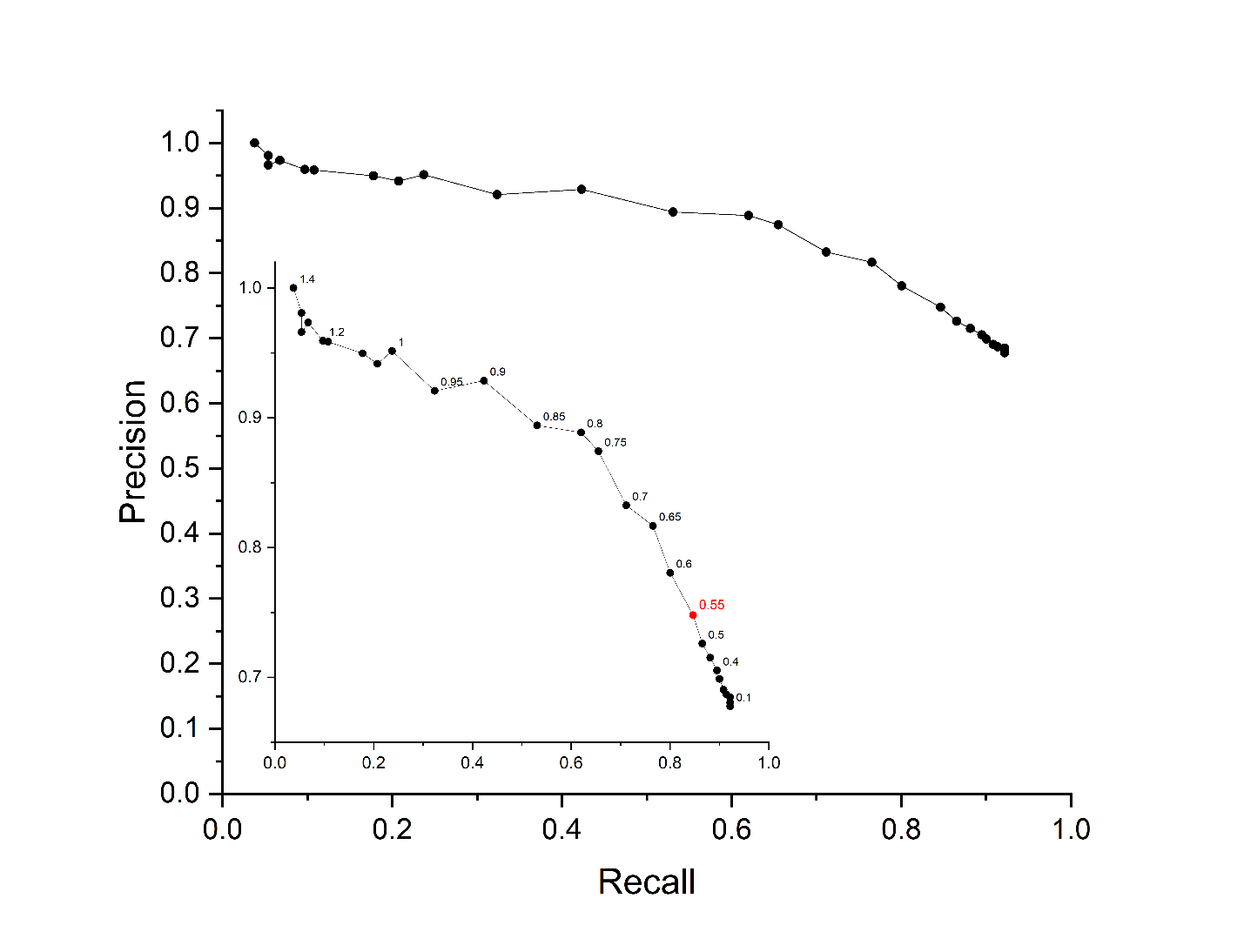


**Figure S1.** Precision vs Recall for predictions computed on the entire structures (i.e. containing all chains for multimers) containing the target sites. Each point is associated with its corresponding scoring function value. Within the figure, a zoomed-in view of the results is shown, focusing on the precision range from 0.8 to 1.0. The scoring function value corresponding to the maximum F1-score is highlighted in red.


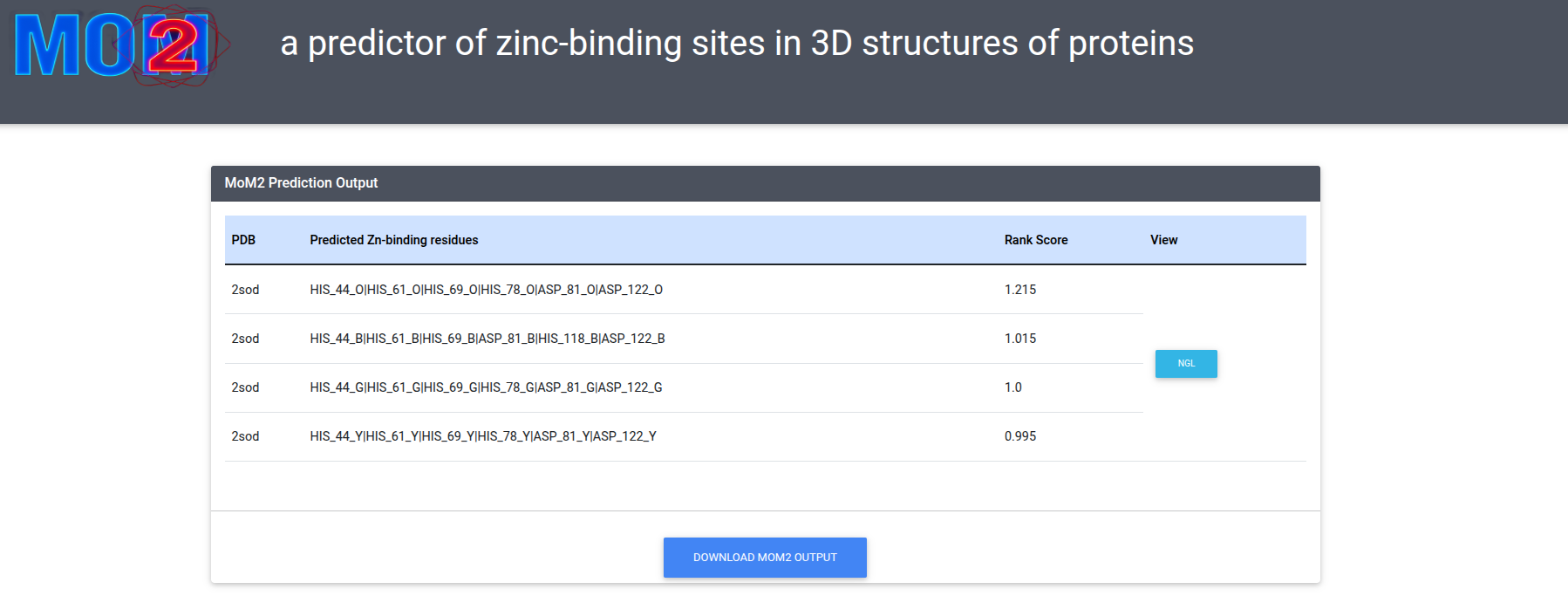


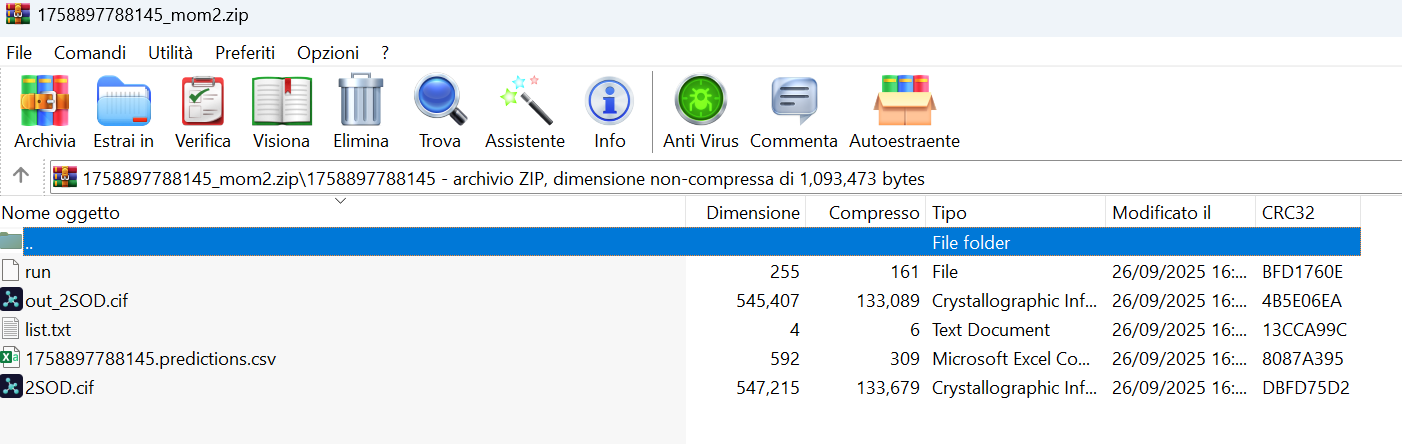


**Figure S2.** Output pages of the MoM2 web server. The csv file reports various parameters produced along the workflow, such as #atoms_in, the number of atoms not pertaining to the ligand residues within 3.0 Å from the predicted metal ion (the lower the better); n_ligands, the number of predicted metal ligands and n_connections, the number of all possible pairwise relationships among the ligand residues.


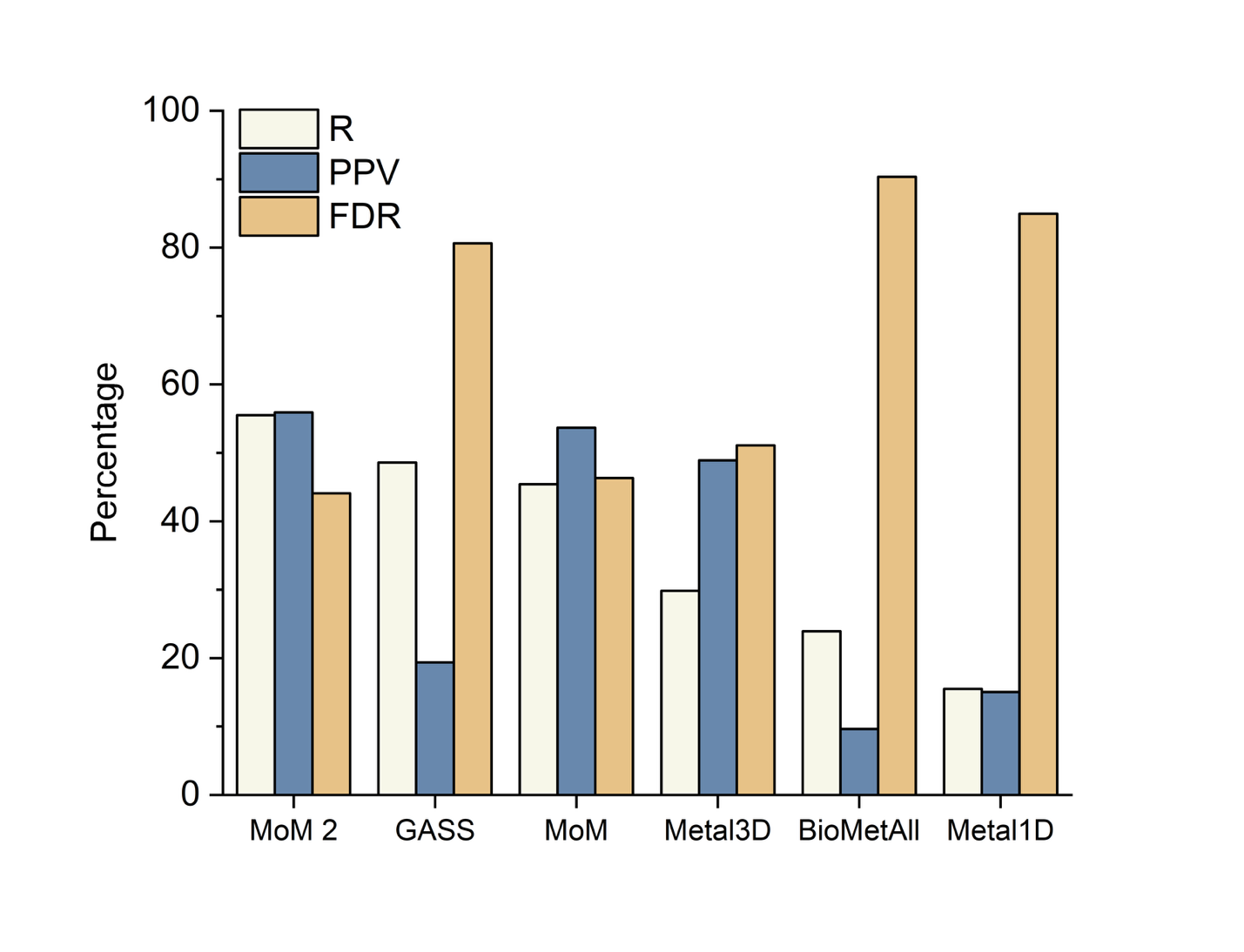


**Figure S3.** Performance of the predictors based on the top 5 outputs. Recall (R), positive predicted value (PPV) and false discovery rate value (FDR) are displayed for all the predictors.

**Table S1. Recall and False Discovery Rate of the 10 neural networks used to predict whether a pair of residues belongs to an MBS**

| **Type of the first ligand** | **Type of the second ligand** | **Recall** | **FDR** |
| --- | --- | --- | --- |
| ASP | ASP | 0.67 | 0.01 |
| ASP | CYS | 0.99 | 0.10 |
| ASP | GLU | 0.99 | 0.14 |
| ASP | HIS | 0.77 | 0.23 |
| CYS | CYS | 0.78 | 0.21 |
| CYS | GLU | 0.99 | 0.11 |
| CYS | HIS | 0.67 | 0.24 |
| GLU | GLU | 0.99 | 0.01 |
| HIS | GLU | 0.91 | 0.24 |
| HIS | HIS | 0.81 | 0.22 |
